# Advancing drug discovery using the power of the human genome

**DOI:** 10.1002/path.5664

**Published:** 2021-04-09

**Authors:** Karl Heilbron, Sahar V Mozaffari, Vladimir Vacic, Peng Yue, Wei Wang, Jingchunzi Shi, Adrian M Jubb, Steven J Pitts, Xin Wang

**Affiliations:** ^1^ 23andMe, Inc. Sunnyvale CA USA

**Keywords:** GWAS, human genetics, therapeutic discovery, drug development, polygenic risk score, direct‐to‐consumer, precision medicine

## Abstract

Human genetics plays an increasingly important role in drug development and population health. Here we review the history of human genetics in the context of accelerating the discovery of therapies, present examples of how human genetics evidence supports successful drug targets, and discuss how polygenic risk scores could be beneficial in various clinical settings. We highlight the value of direct‐to‐consumer platforms in the era of fast‐paced big data biotechnology, and how diverse genetic and health data can benefit society. © 2021 23andMe, Inc. *The Journal of Pathology* published by John Wiley & Sons, Ltd. on behalf of The Pathological Society of Great Britain and Ireland.

## From non‐clinical models to human genetics

All drugs entering human trials have shown evidence of efficacy in non‐clinical models of disease, and yet a large fraction fail to demonstrate efficacy in humans. Of phase II trials conducted between 2005 and 2015, 51% failed to achieve their prespecified primary objective [1]. Within AstraZeneca from 2005 to 2010, lack of efficacy was responsible for the closure of 57% of phase IIa projects and 88% of phase IIb projects [2]. Clearly, efficacy in treating non‐clinical disease models is not always an adequate proxy for efficacy in treating human disease. Human genetic studies take advantage of naturally occurring genetic variations that may mimic the effect of therapeutically perturbing a gene. Unlike studies of animal or *in vitro* models, human genetic studies are well‐suited to the task of establishing a relationship between human disease and variation in the activity of a potential drug target or pathway, thereby decreasing the probability that a drug trial will fail due to lack of efficacy [3].

When the draft human genome was published in 2001, authors from the International Human Genome Consortium wrote: ‘Knowing the complete set of human genes and proteins will greatly expand the search for suitable drug targets. Although only a minority of human genes may be drug targets, it has been predicted that the number will exceed several thousand, and this prospect has led to a massive expansion of genomic research in pharmaceutical research and development’ [4]. Initial efforts were focused on identifying the consensus sequence of all genes that were homologous to existing drug targets and all druggable genes so that they could be tested for therapeutic potential, but the effect of genetic variation on gene function or activity has since come to play a much larger role in the field.

Genetics‐driven drug discovery has had notable successes for Mendelian disorders (see Glossary of terms), in which rare genetic variants have large effects on the function of a single gene. Examples include enzyme replacement therapies for lysosomal storage diseases [5] and nusinersen for spinal muscular atrophy [6]. Many of the diseases that cause the greatest global morbidity and mortality also have Mendelian subtypes. For example, about 11% of early onset Alzheimer's disease cases are due to mutations in *APP*, *PSEN1*, and *PSEN2* [7]. Nelson *et al* [8] found that drugs were about 7.2 times more likely to be approved if the drug's target was linked to a Mendelian form of the disease for which the drug was indicated. Follow‐up work by King *et al* [9] also estimated that the odds of approval were more than six times higher given Mendelian genetic support. With the advancement of sequencing technologies, more rare genetic causes of common diseases have been discovered [10, 11, 12, 13, 14, 15, 16]. The increasing number of whole‐exome and whole‐genome sequences will further shed light on the low‐frequency end of the spectrum of human genetic variation (e.g. The 1000 Genomes Project [17]; Haplotype Reference Consortium [18]; The Genome Aggregation Database [19, 20]; and Trans‐Omics for Precision Medicine program [21]).

However, for the vast majority of highly prevalent diseases, the heritable risk is driven by a large number of common variants (often in the form of single nucleotide polymorphisms, i.e. SNPs, see Glossary of terms) with much smaller individual effect sizes [22]. This finding comes as a result of the widespread application of genome‐wide association studies (GWAS, see Glossary of terms) to scan the genome to look for associations of genetic variants with disease risk. Nelson *et al* [8] and King *et al* [9] investigated whether genetic support from GWAS was predictive of drug approval. Retrospectively, they found that drugs with GWAS support were at least two times more likely to be approved, particularly if the GWAS signal appeared to be driven by a mutation that altered the amino acid sequence of the gene product [8, 9].

## The era of human genetics‐driven drug discovery

Increasing focus on human genetics by academia and industry has caused the number of genetic associations recorded in the GWAS Catalog (https://www.ebi.ac.uk/gwas/) to expand rapidly in the past few years, providing novel leads for genetics‐driven drug discovery. This growth will probably continue, given the availability of large and diverse databases of genotyped individuals, such as The China Kadoorie Biobank (www.ckbiobank.org), Biobank Japan (http://jenger.riken.jp/en/), the UK Biobank (https://www.ukbiobank.ac.uk/), the Million Veteran Program (US Department of Veterans Affairs, https://www.research.va.gov/mvp/), the All of Us Research Program (NIH, Bethesda, MD, USA, https://allofus.nih.gov/), and direct‐to‐consumer databases. In addition, several countries with single‐payer healthcare systems (such as Denmark, Estonia, Finland, Iceland, and The Netherlands) have established national biobanking infrastructure and large‐scale population genotyping initiatives [23].

The number of individuals who volunteer their data through various platforms for advancing biomedical research has led to substantially larger genetic studies than would have been possible otherwise. In 2016, the largest GWAS meta‐analysis at the time was published on major depressive disorder [24]. In 2018, genetic analyses were conducted in over 1 million individuals for blood pressure traits [25]. In 2019, a meta‐analysis of tobacco and alcohol use and a meta‐analysis of insomnia included approximately 1.2 million and over 1.3 million individuals, respectively [26, 27].

One approach to increase the power of GWAS for drug discovery is to scale participation through direct‐to‐consumer platforms. Conventional biobanks create repositories of biospecimens from recruited participants that are later analyzed. Under the direct‐to‐consumer model, customers' DNA is genotyped and analyzed to provide insights regarding their ancestry, health risks, and other traits that are influenced by genetics. These customers may then volunteer their genetic and phenotypic data for research purposes, engaging and empowering a wide range of participants. Today, 23andMe, Inc. (http://www.23andme.com), a direct‐to‐consumer genetics company established in 2006, has a database that includes more than 12 million customers. Approximately 80% of the customers actively opt in and consent to research and have contributed over 3 billion phenotypic data points. Genealogy companies with large customer bases, such as MyHeritage, have also recently expanded to include DNA testing and health products, and have significant potential to grow in scale.

**Figure 1:**
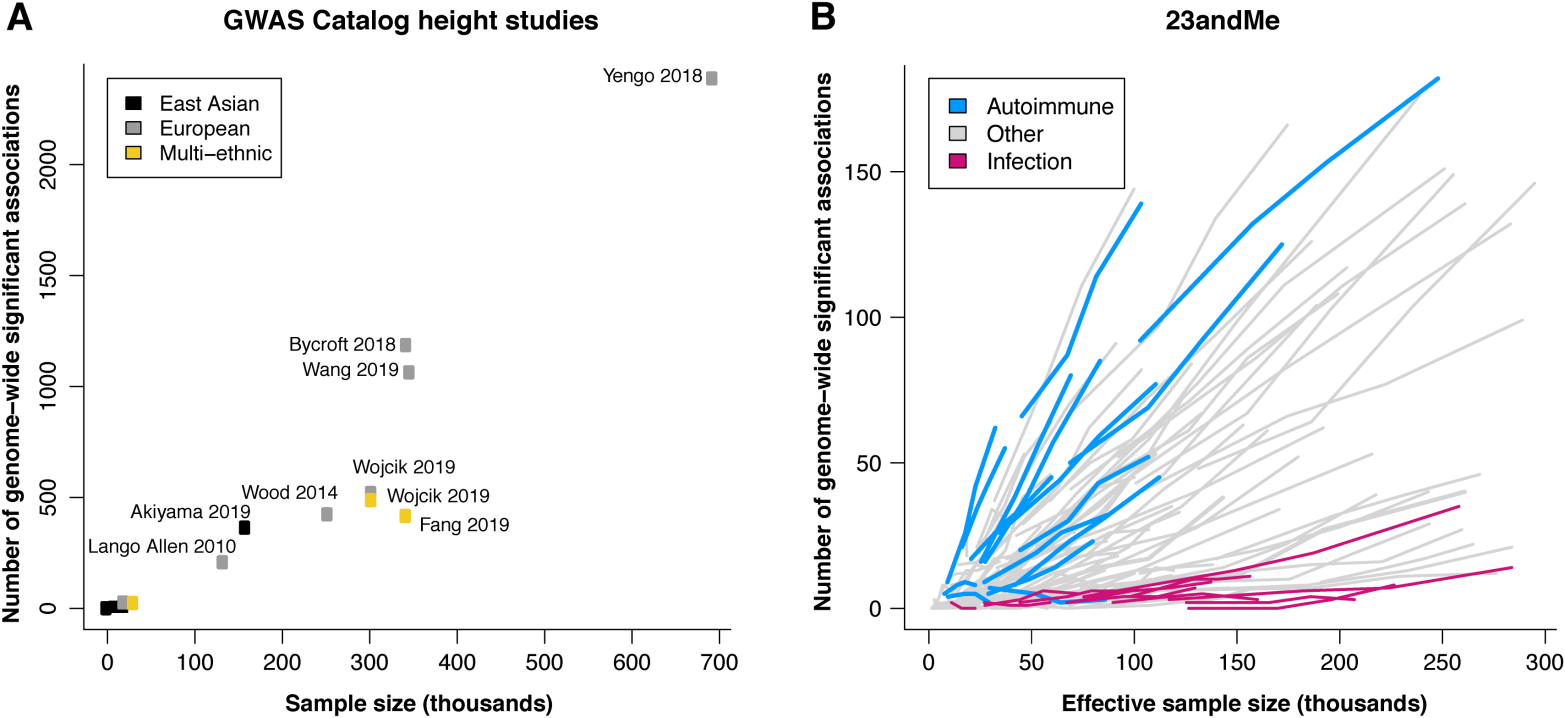
The number of genome‐wide significant loci discovered increases linearly as a function of sample size. (A) The number of genome‐wide significant loci discovered as a function of sample size for ‘body height’ GWAS recorded in the GWAS Catalog as of 1 November 2020 (see supplementary material, Table S1 for details of the studies used). The associated publication for each study was manually assessed, excluding (1) GWAS of traits other than adult height, (2) GWAS of individuals of European ancestry with fewer than 19 000 cases, and (3) GWAS conducted using whole‐genome or whole‐exome sequencing data. SNPs with *p* > 5 × 10^−8^ and SNPs that were only identified by conditional analysis were also excluded. The color of the points represents the ancestry of the individuals included in the study (black = East Asian; gray = European; gold = multi‐ethnic). (B) Trajectories for a selection of GWAS for 126 23andMe disease phenotypes conducted in individuals of European ancestry at four time points between October 2017 and August 2019. Effective sample size is defined as N_eff_ = 4/(1/N_cases_ + 1/N_controls_) for binary phenotypes and is equal to the sample size for continuous phenotypes. Trajectories for autoimmune diseases and infection phenotypes are highlighted in blue and pink, respectively.

Within the GWAS catalog, studies on adult height as a model polygenic trait have achieved some of the largest sample sizes and continue to grow considerably over time. The number of independent risk loci identified for height has grown proportionally to the increase in sample size (Figure 1A), previously also observed in Panagiotou *et al* [28]. A similar trend is seen in the 23andMe database across a wide range of disease phenotypes (Figure 1B). Even with very large sample sizes, we anticipate that the availability of large‐scale genotyped cohorts will continue to yield approximately proportional increases in the number of discovered GWAS associations. Larger study cohorts often correlate with greater discovery power and, therefore, should accelerate therapeutic target discovery. GWAS is powered to find associations that explain the largest proportion of phenotypic variation first. As sample sizes increase, the individual effect sizes of the newly discovered associations will probably be smaller, or allele frequencies lower [29]. Even such, these associations may drive new therapeutic hypotheses as the effect of the allele in the population usually differs from the therapeutic effect of a drug (e.g. statins). For fine mapping, van de Bunt *et al* [30] showed, via simulation and empirical data, that the sizes of credible sets, defined as the minimum set of variants 95% likely to contain the causal variant [31], negatively correlate with the power to detect association signals, thereby increasing the confidence in identifying a causal variant or gene. Meta‐analyses are now regularly employed to achieve larger study sample sizes. Additionally, heterogeneity analysis may identify possible false‐positive findings due to biases originating from single studies and serve as some level of replication [32] to further boost the confidence in therapeutic hypotheses.

Whereas phenotyping of individuals in cohorts derived from health care systems may be performed by both computational analysis of electronic medical records and data that are self‐reported via web‐ or smart phone‐based questionnaires, direct‐to‐consumer companies primarily rely on the latter. Self‐reporting has proven to be an effective method to collect health and medically relevant data at scale. A proof‐of‐concept study showed 100% concordance between self‐reported Parkinson's diagnosis and neurologist assessments in 50 patients [33] and an early set of GWAS based on self‐reported medical phenotypes was able to replicate 75% of National Human Genome Research Institute (NIH)‐curated genetic associations [34]. A two‐stage GWAS design that used self‐reported data in the discovery phase and clinically ascertained patients in the replication phase has further validated the use of ‘self‐reported data as a platform for discovery’ [35].

Self‐reported phenotypes are imperfect. For example, numerical laboratory values are not well‐suited for self‐reporting. These phenotypes may suffer from both reporting of misdiagnoses (e.g. mild cases of eczema versus psoriasis) and incorrect reporting of diagnoses (e.g. osteoarthritis versus rheumatoid arthritis). Whereas the latter can be mitigated by asking follow‐up questions and aggregating answers to several related questions, the former will be a much greater challenge. The construction of accurate disease phenotypes from medical records also has its difficulties, as diagnoses may only be present in the unstructured text of clinical notes or in the form of billing codes justifying tests or procedures that are later rejected with additional information [36]. In the case of both electronic medical record‐based phenotyping and self‐reporting, these potential shortcomings are typically offset by the scalability and speed of data collection for GWAS purposes, where scale can be a dominant factor for discovery. As a testament to the validity of the self‐report approach, the UK Biobank has also adopted self‐reporting for data collection, in addition to the use of medical records. However, as a result of either misdiagnosis or misreporting, the potential non‐specificity of the association between a locus and a disease will need follow‐up confirmation [37]. A recent analysis using UK Biobank data compared GWAS using cases derived via hospital records versus those via verbal questionnaires. Importantly, the study examined variants beyond previous replication studies that focused mostly on genome‐wide significant associations. They found high genetic correlations (>0.8) for 27 of 41 phenotypes studied and showed that combining the two phenotyping methods does not significantly alter GWAS effect size estimates. The increase in sample size by leveraging both phenotyping methods improved the power of identifying alleles associated with disease risk. Hence, utilizing self‐reported data together with structured hospital records can enhance human genetics studies [38].

A disproportionate number of published GWAS so far have focused on individuals of European descent [39, 40, 41, 42]. As of 2018, fewer than 20% of study participants in the GWAS catalog were non‐European, despite making up greater than 80% of the global population [43]. To increase the understanding of human diversity and to improve on health equality, establishing study cohorts from under‐represented populations is critical. Individuals of European descent represent only a limited fraction of the total human genetic variation. Studies in populations with African and/or Latino ancestry tend to find a greater number of genetic associations when compared with studies in an equivalent number of European‐ancestry individuals [44]. Diverse cohorts represent unique opportunities for identifying novel drug targets based on genetic variants that are less frequent or even absent in people of European ancestry. Multiple *APOL1* gene variants that are specific to African Americans were found to be associated with chronic kidney disease [45, 46]. Many diseases have greater prevalence in non‐Europeans. For example, according to the most recent data from the US Centers for Disease Control and Prevention (https://www.cdc.gov/asthma/most_recent_national_asthma_data.htm), Puerto Rican children are two to four times more likely to have asthma compared with non‐Hispanic Whites [47]; data from the National Institute of Diabetes and Digestive and Kidney Diseases (https://www.niddk.nih.gov/health-information/kidney-disease/race-ethnicity) show that African Americans are four times more likely to have end‐stage kidney disease compared with Americans of European ancestry [48]. Genetic discoveries will have greater discovery power in populations where a disease is more prevalent and, hence, with larger disease cohorts; at the same time, these discoveries will be more relevant and be beneficial for these populations.

Improving participation and recruitment is one important avenue for increasing the ethnic diversity of human genetic studies [49, 50], and where very large genetic cohorts can play a vital role. For example, although the majority of the 23andMe customer base is made up of individuals of predominantly European ancestry (73%), given the large number of research participants, even relatively smaller Latino (12%) and African‐American (4%) cohorts are among the largest in the world. As of 2019, among those who have consented to participate in research, the 23andMe database included over 300 000 African‐American individuals, compared with approximately 148 500 (18% of approximately 825 000) veterans enrolled so far in the Million Veteran Program (2019) [51, 52] or approximately 46 000 (20% of approximately 230 000) participants enrolled in the NIH All of Us study cohort (2020) [53]. 23andMe launched the African genetics project in 2016 and the Global Genetics Project was launched in early 2018 to recruit customers from under‐represented countries.

Studies of populations with historically small population sizes (e.g. Iceland's deCODE database [https://www.decode.com/] and Finland's FinnGen research project [https://www.finngen.fi/en/]) and cohorts with a high rate of consanguinity (e.g. the Pakistan Risk of Myocardial Infarction Study [54], https://www.phpc.cam.ac.uk/ceu/promis/) also offer unique opportunities for therapeutic discovery. deCODE genetics was acquired by Amgen in 2012 [55], and FinnGen currently has 12 industry partners [56]. Strongly deleterious mutations that disrupt gene function may persist at higher frequencies in smaller populations and provide insights into the function of human genes. As such, some of the genetic variants with the largest effect sizes have been identified in cohorts with unique population structures [57, 58, 59], with *PCSK9* being an example [60]. One limitation of these cohorts is that they only have access to the genetic variation within the population. If these populations are bottlenecked, then they will present limited opportunities for understanding the full spectrum of human genetic diversity.

Recognizing the untapped potential of human genetics, the biotechnology and pharmaceutical industries have had a longstanding interest in investing in large genomics initiatives, consortia, and databases in order to accelerate drug discovery efforts. Below we illustrate a variety of examples of this investment since the Human Genome Project (https://www.genome.gov/human-genome-project). In 2007, the Genetic Association Information Network (GAIN) collaborative research group was established as a public–private partnership in order to ‘investigate the genetic basis of common diseases’ [61]. In the following years, a large number of industry‐funded studies found genes linked to different diseases, such as schizophrenia and type II diabetes [13, 62]. The Global Alliance for Genomics and Health (https://www.ga4gh.org/) formed in 2013 to accelerate research and medicine, with a specific mission to foster ‘effective and responsible data sharing’. In 2014, OpenTargets [63] was established as a public–private consortium that integrates the wealth of data from publicly available genomic resources to enhance the ability to systematically identify and prioritize drug targets. In 2018, Genomics plc and Vertex Pharmaceuticals signed a 3‐year contract to use machine learning and human genetics in target discovery and precision medicine [64]. In the same year, GlaxoSmithKline plc (GSK) entered into a collaboration with 23andMe Inc. to leverage human genetics for the discovery of novel medicines [65]. More recently, several companies, including Regeneron, AbbVie, Alnylam, AstraZeneca, Biogen, and Pfizer, have invested in the UK Biobank exome sequencing initiative to accelerate data generation [66, 67].

## Human genetics can identify successful drug targets

Many successful drug targets were first identified as a result of genetic associations. For example, gain‐of‐function variants in *PCSK9* were first discovered in 2003 in French families with high rates of heart disease, suggesting that this gene may play a causal role in cardiovascular risk [60]. Cohen *et al* [68] later found that a loss‐of‐function mutation in *PCSK9* correlated with significantly lower plasma cholesterol levels in 2% of African‐Americans in the Dallas Heart Study. Spurred on by these associations, the first PCSK9 inhibitors were approved by the FDA to lower LDL cholesterol levels in 2015 (alirocumab and evolocumab) [69, 70] and to prevent heart attack and stroke in 2017 (evolocumab) [71], thereby improving cardiovascular outcomes.

**Figure 2:**
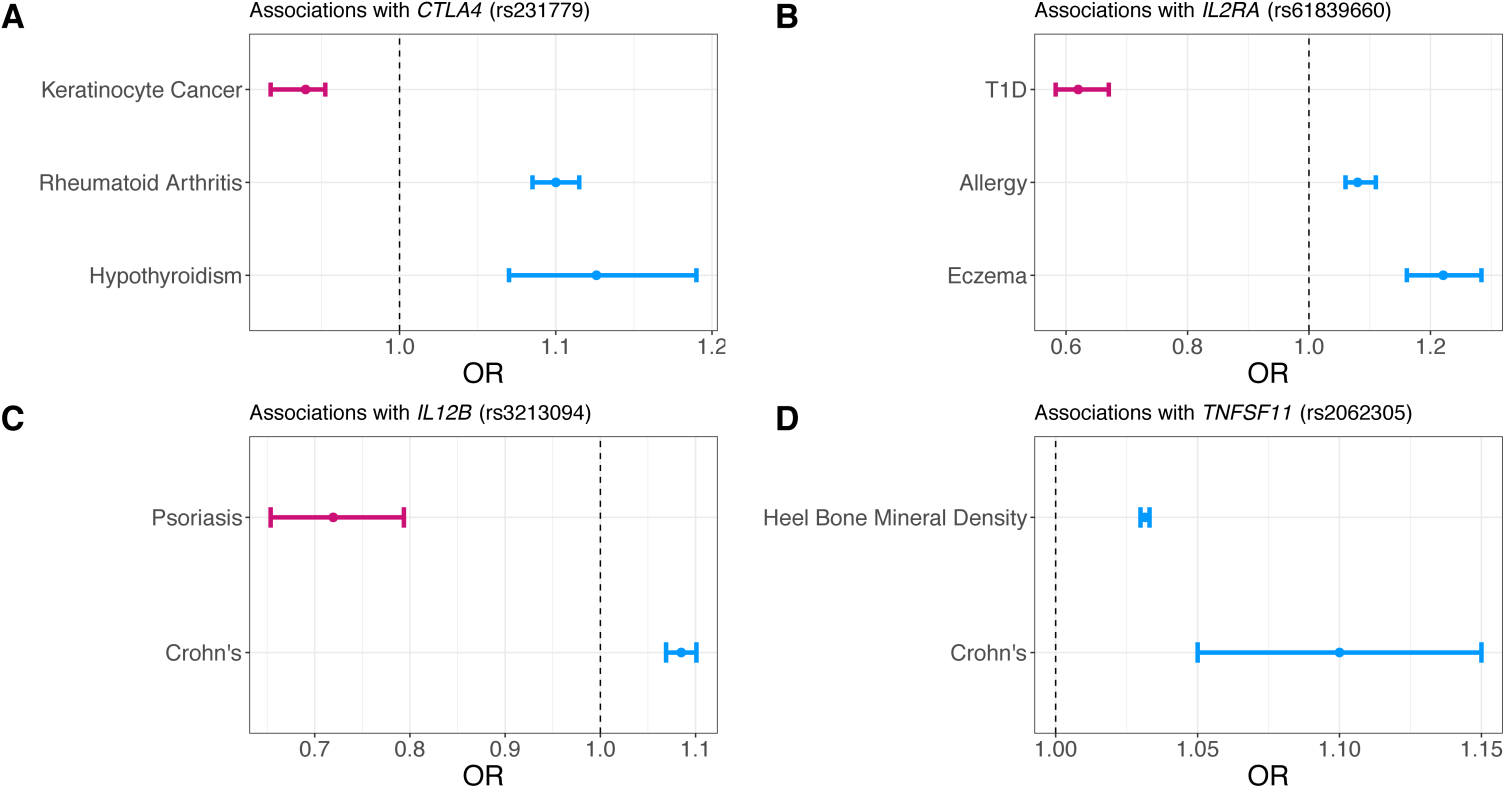
Effect sizes for variants in four genes from OpenGWAS and GWAS Catalog. Odds ratios (OR) and 95% confidence intervals for four gene–disease indication sets are shown. Colors represent directions of association (pink: OR < 1, blue: OR > 1). Effect sizes are for (A) rs231779 (*CTLA4*) in hypothyroidism, rheumatoid arthritis, and keratinocyte cancer; (B) rs61839660 (*IL2RA*) in type I diabetes (T1D), allergy, and eczema; (C) rs3213094 (*IL12B*) in psoriasis and Crohn's disease; (D) rs2062305 (*TNFSF11*) in heel bone mineral density and Crohn's disease, in the European population. Association summary statistics are accessed via the GWAS Catalog and OpenGWAS API [79, 80].

Human genetics can also retrospectively identify important features of successful drug targets. Cancer immunotherapies activate the immune system to recognize and kill tumors [72]. Variants in some immunotherapy targets show risk associations in opposite directions for cancer and immune phenotypes. This suggests that boosting the immune system could reduce cancer risk and that it may be possible to identify novel immunotherapies by screening for similar types of genetic associations. For example, CTLA4 is an immune checkpoint for T‐cell activation and is the target for ipilimumab and tremelimumab. Genetic variants near this gene are associated with an increased risk of immune phenotypes, including thyroid diseases [73, 74, 75], rheumatoid arthritis [76], and type I diabetes [77], but are also associated with a decreased risk of multiple skin cancers [78] (Figure 2A). Recognition of the potential of this cancer‐autoimmunity signature may help to identify the pivotal nodes in the vast interconnected network of the human immune system to increase the likelihood of clinical success for future therapies.

Genetic associations have been able to successfully predict drug side‐effects and drug repurposing opportunities. Basiliximab is an immunosuppressant that is used to prevent transplant rejection. It is a monoclonal antibody targeting the gene product of *IL2RA* but has been shown to increase the risk of diabetes [77, 81]. Variants near *IL2RA* show genetic associations with various immune phenotypes [82, 83, 84], as expected for an immunosuppressant, but also for type I diabetes [77, 81] (Figure 2B). Topiramate, an anticonvulsant used to treat epilepsy and prevent adult migraines, was later shown to be effective in chronic weight management [85, 86]. Topiramate targets the gene product of *SCN1A*. Genetic variants near *SCN1A* are associated with epilepsy [87] and body mass index [88, 89]. Topiramate has been shown retrospectively to be an unsuccessful treatment for inflammatory bowel disease (IBD) [90]. Although it has been suggested that a well‐designed and powered clinical trial could show that topiramate is effective for IBD, there is no association of *SCN1A* with IBD in the GWAS catalog (with approximately 29 000 cases in the largest study cohort) [91]. Ustekinumab is an anti‐IL12B monoclonal antibody used to treat psoriasis [3, 92] and has since been successfully repurposed to treat Crohn's disease [93, 94]. Genetic variants near *IL12B* are associated with both psoriasis [95] and Crohn's disease [84] (Figure 2C). Denosumab, a monoclonal antibody against TNFSF11, is used to treat osteoporosis. Franke *et al* [96] subsequently found that variants near *TNFSF11* were also associated with Crohn's disease, ultimately leading to denosumab being successfully repurposed for Crohn's disease [85, 93, 97]. *TNFSF11* variants are associated with both heel bone mineral density [98, 99] and Crohn's disease in multiple studies (Figure 2D).

In summary, human genetics has prospectively identified successful drug targets, is often able to retrospectively recapitulate the genetic profile of successful drugs informing future development efforts and relevant toxicities, and can provide evidence for opportunities to repurpose existing drugs.

## Polygenic risk scores in precision medicine

The era of human genetics‐driven drug discovery is an exciting time, not only for gene‐focused efforts, but also for advancing precision medicine. Most common diseases are driven by a complex genetic architecture that involves a large number of genetic variants. The cumulative effect of these genetic variants is informative of an individual's overall risk of disease and could help to personalize treatment and preventative measures. To that end, polygenic risk scores (PRS) combine the risk effects from many genetic variants and have been widely used to predict disease risk [100, 101].

PRS applied in clinical settings can improve disease diagnosis and the prediction of health outcomes. Many studies demonstrated the potential of PRS to predict risks of individuals and improve risk stratification for different diseases, such as Alzheimer's disease [102], ischemic stroke [103], and skin cancer [104]. Relative to monogenic mutations, PRS can identify a larger fraction of the population that is at high disease risk and are thus potentially more clinically relevant. A PRS constructed for cardiovascular disease can identify up to 20‐fold more people at comparable or greater risk than those identified with only the known monogenic mutations [105]. For some diseases, PRS have been able to further stratify risks on top of known genetic risk variants, such as in *BRCA1* and *BRCA2* for breast cancer [106, 107, 108], in *MSH2*, *MLH1*, *MSH6*, and *PMS2* for Lynch syndrome [108], and in *APOE* for Alzheimer's disease [109]. PRS also show great promise as a tool in refining disease diagnosis. It is particularly challenging to accurately diagnose diseases with similar symptoms or to diagnose diseases that progress slowly. Knevel *et al* [110] reported that adding PRS of different inflammatory diseases to existing clinical information can improve correct diagnosis at the first visit from the initial 39% to 51% (McFadden's R^2^, see Glossary of terms).

With the potential ability to better stratify risk and identify disease subtypes, and, therefore, better enrichment of patient populations, PRS shows great promise for clinical trials. Traditional trial designs compare the effects of the treatment relative to a placebo within a typically homogenous patient population. Inherent patient heterogeneity can lead to challenges due to insufficient biomarkers or outcome measures [111]. PRS that are disease subtype‐specific may better capture the clinical heterogeneity among individual patients, including their response to available treatments, development of complications, and rate of disease progression. In fact, partitioned PRS have been proposed as a promising tool to capture disease subtypes in type II diabetes [112]. In amyotrophic lateral sclerosis, identifying fast progressing patients in a lead‐in period was shown to have the potential to shorten clinical trials, and result in cost and time savings [113]. For diseases such as non‐alcoholic steatohepatitis, there are currently no approved therapies, despite significant clinical and economic burden. In addition to searching for better drug targets [114], selecting the faster progressors within non‐alcoholic steatohepatitis patients may be a key to successful trials, which have been long and often complicated by high placebo responses [115].

To define appropriate patient populations for successful drug development and use, identifying accurate, predictive biomarkers may be pivotal. We are still at the very early stage of applying PRS to predict a patient's response against a given therapy, but there have been some early successes in cardiovascular and neurological diseases. Statin therapy was shown to lead to greater risk reduction in those with high genetic risk for the first coronary event [116]; and a high PRS for coronary artery disease (>90th percentile) was associated with a greater reduction (37% versus 13%) in major adverse cardiovascular events compared with a lower PRS (≤90th percentile) upon treatment with alirocumab/anti‐PCSK9 [117]. Recently, a PRS constructed for migraine was able to identify subgroups of individuals with a higher likelihood of responding to triptans when looking for associations between migraine PRS and migraine‐specific drug efficacy [118].

The potential for PRS to predict response to therapy could have large impacts on clinical trials. Treatment of cancer patients with PD1/PD‐L1 checkpoint inhibitors has been associated with immune‐related adverse events, most commonly in skin. Furthermore, the development of these adverse events is associated with longer overall survival. Consistent with the role of immune checkpoints in self‐tolerance and autoimmunity, Khan *et al* [119] set out to apply PRS constructed for skin autoimmunity (psoriasis, vitiligo, atopic dermatitis) to a failed phase III clinical trial that tested the efficacy of the immune checkpoint inhibitor atezolizumab/anti‐PD‐L1 (CD274) as a bladder cancer treatment. High skin autoimmunity polygenic risk individuals had longer overall survival, making the PRS predictive of the treatment effects. Future trials are needed to test whether selecting individuals whose genetics predicted a high likelihood of response will lead to a successful trial [119].

## Discussion

Non‐clinical models of disease play a critical role in target validation and the screening of drug candidates. However, the efficacy of a drug in a non‐clinical model does not always translate into efficacy in patients. Human genetic data can serve as a complementary tool to increase confidence that modulating a target is likely to improve patient outcomes. In this regard, GWAS have been successful in identifying variants and genes associated with many human diseases, helping us to understand their biological underpinnings and informing drug discovery efforts that we anticipate will have a higher likelihood of clinical success.

Many diseases have both rare and common genetic risk factors. Rare variants in a gene can lead to Mendelian forms of a disease, whereas common variants affecting the same gene can influence non‐Mendelian disease susceptibility. For example, the *LRRK2* p.G2019S variant confers an approximately 25% lifetime risk of Parkinson's disease (minor allele frequency = 0.15%, odds ratio = 11.3 in Europeans), whereas a common variant (rs76904798, minor allele frequency = 14.4% in Europeans) that is linked to a *LRRK2* expression quantitative trait locus (eQTL, see Glossary of terms) is associated with an odds ratio of 1.15 [120]. Having multiple variants in a locus that influence a disease creates an allelic series, which can potentially demonstrate that larger perturbations of gene function lead to larger effects on disease susceptibility [3]. These dose–response curves are an important aspect when establishing a causal relationship between gene function and disease [121], and show how GWAS can build upon established, high‐penetrance genetic links to disease to inform disease pathology in ‘idiopathic’ subsets.

Case–control GWAS of disease phenotypes conventionally identify genetic variants associated with lifetime susceptibility. With increasingly large cohorts and availability of diverse study populations, GWAS that focus on disease severity and progression may reveal further opportunities for novel therapies [122]. As societal disease burden increases due to an aging population, treatments to slow disease progression and to lessen the effects of a disease are in need. However, the use of GWAS in drug discovery and development has a number of limitations. For example, perturbing pathways and gene functions that influence developmental processes may not make for effective therapies in adults. Drug discovery that is informed by human genetics is also not equally applicable to all disease areas. Medicines to combat infectious diseases and new antibiotics are highly unlikely to be derived from GWAS (Figure 1B). Host–microbial interactions, rapid selection, and drug resistance are all factors that play a large role in the effectiveness of these treatments that are not easily captured in genetic studies. However, genetic susceptibility may still prove useful for understanding variation in infection rates, symptoms, and response to therapy [123, 124, 125, 126]. Moreover, intrinsic differences in genetic architecture may explain why some phenotypes yield significantly more genetic associations than others for a given sample size (Figure 1B). These include differences in heritability, polygenicity, and the distribution of effects and allele frequencies of causal variants.

Most GWAS associations are in non‐coding regions, some of which have been shown to influence disease risk via regulating gene expression [127]. The increasing availability of large functional datasets and genomics resources, such as the Encyclopedia of DNA Elements (ENCODE) project [128] and the Genotype‐Tissue Expression (GTEx) project [129], have advanced the functional annotation of these variants. However, causal gene identification and linking causal genes to function remain challenging. With the availability of genome editing tools, such as zinc finger nucleases (ZFNs), transcription activator‐like effector nucleases (TALENs), and CRISPR/Cas systems (Nobel Prize in Chemistry, 2020) [130], we are now able to perturb the entire genome with unprecedented scale and fine control. Functional genomics screens with phenotypic assay readouts are a promising avenue that can deconvolute this complexity. Some cancer types have been the first to benefit from these screens, as fitness and survival of tumor cells are relatively straightforward phenotypic readouts. *Ptpn2* was identified by an *in vivo* CRISPR screen as a promising target to increase the efficacy of immunotherapy [131]. The Wellcome Trust Sanger Institute [132] and the Broad Institute [133] later prioritized Werner syndrome RecQ helicase as a key survival gene and an attractive drug target in tumors characterized by high microsatellite instability. Although they were not initially discovered from GWAS, these examples reveal the potential of such an approach. In addition to knock‐out screens where a gene is disrupted and hence gene function ablated, knock‐in assays that rely on the less‐efficient homology‐directed repair to introduce precise changes to the DNA sequence are more challenging. Gupta *et al* [134] utilized both deletion and base editing to link a GWAS‐identified SNP to a distal regulation mechanism in five cardiovascular diseases. However, most GWAS associations are not resolved to a single variant due to linkage disequilibrium (LD, see Glossary of terms) [135], complicating the identification of candidate causal variants for functional follow‐ups and underscores the value for genome‐wide knock‐in screens. Recently, methods were developed to screen transcriptional or splicing variants endogenously [136] and to perform high‐throughput screens using base editors [137], greatly increasing the scalability of functional genomics assays. Additional methods, such as CRISPR‐QTL [138] and TAP‐seq [139], have expanded CRISPR's potential by mapping enhancer–gene pairs. These innovations may further enable linking GWAS associations to genes and their functions and potentially offer new therapeutic modalities for genes that are not easily targeted with current approaches.

PRS are a promising tool for precision medicine. Many studies have shown that PRS has great potential for improving diagnosis, prediction of health outcomes, response to therapy, and clinical trials. Validated PRS can also impact individual behaviors, clinical decision making, as well as implementation of population screening strategies. For example, research shows that polygenic risk influences the penetrance of monogenic disease risk factors [105, 108], indicating the utility of PRS in counseling and clinical decision making for carriers of pathogenic variants. In a recent study, Forgetta *et al* [140] was able to use PRS for quantitative ultrasound speed of sound at the heel, a heritable risk factor for osteoporotic fracture, to identify low‐risk individuals who can be safely excluded from an expensive fracture risk screening.

However, PRS is limited by disease heritability, and genetics generally contributes less than the environment to overall phenotypic variation. Future risk models will probably need to incorporate both genetics and environmental factors to be of maximal predictive value. In the short term, assessing PRS alongside existing risk factors (such as age and sex) will be important for understanding their clinical utilities. Recently, a genetic risk score of coronary heart disease was shown to have minimal value in improving risk stratification to predict incident events compared with a guideline‐based risk equation [141].

One of the main limitations of many PRS studies is that they are carried out retrospectively. In order to validate these PRS, more rigorous and prospective studies are needed to replicate the results, including randomized controlled clinical trials. Another limitation in establishing the clinical utility of PRS is to ensure they are applicable across diverse populations, especially under‐represented groups. Due to the vast over‐representation of European‐ancestry individuals in GWAS studies, the majority of PRS are generated using European‐based associations and tend to have attenuated prediction accuracy when applied to non‐European populations [142]. Consequently, the clinical application of PRS is currently most suitable to a small proportion of the global population. Substantial investments in methodology development and research infrastructure improvements are needed to achieve transferability of PRS across diverse populations, and to ensure thorough exploration of the value of PRS within clinical settings. The ability to create predictive polygenic models requires large training cohorts, both to identify genetic variants associated with a disease and to estimate their joint contribution to risk [143]. Large‐scale and diverse databases and biobanks, including direct‐to‐consumer platforms, are in a unique position to develop better, more transferable PRS.

In conclusion, public and private investment in human genetics to date has improved our understanding of human health and will continue to play an important role in drug development. Continued investment to scale these efforts, refine phenotypes, improve computational methods, and increase the diversity of the individuals being studied is essential if we are to fully leverage the human genome and ensure that the products of this research benefit the full breadth of humankind.

## Glossary of terms

**Common variant**

A variant (most often a SNP) with a minor allele frequency of at least 1%.

**Expression quantitative trait loci (eQTL)**

Genomic loci that explain variation in the expression level of mRNAs. An expression trait is the amount of an mRNA transcript for a protein. Chromosomal loci that explain variance in expression traits are called eQTL(s).

**Genome‐wide association study (GWAS)**

An approach used in genetics research to associate genetic variations with disease risk. The method involves scanning the genomes from many different people and looking for genetic markers that can be used to predict the presence of a disease. Once such genetic markers are identified, they can be used to understand how genes contribute to the disease and develop better prevention and treatment strategies.

**Linkage disequilibrium (LD)**

The non‐random association of alleles at different loci in a given population. Loci are said to be in linkage disequilibrium when the frequency of association of their different alleles is higher or lower than what would be expected if the loci were independent and associated randomly.

**McFadden's R** ^**2**^

A measure of explained variation, defined as 1 – log(L_current_)/log(L_null_), where L_current_ denotes the maximum likelihood value from the current fitted model and L_null_ denotes the maximum likelihood value from the null model with only an intercept and no covariates.

**Mendelian disorder/disease**

A disorder/disease that is controlled by a single locus in an inheritance pattern. In such cases, a mutation in a single gene can cause a disease that is inherited according to Mendel's principles.

**Single nucleotide polymorphism (SNP)**

Substitutions of a single nucleotide at a specific genomic location.

## Author contributions statement

KH, SVM, VV, PY, WW, JS, AMJ, SJP and XW conceptualized and wrote the manuscript. KH, SVM and XW generated the figures and table. All authors approved the final version of the manuscript.

## Supporting information


**Table S1.** Height GWAS used in Figure 1A

## References

[1] Wong CH , Siah KW , Lo AW . Estimation of clinical trial success rates and related parameters. Biostatistics 2019; 20: 273–286. 29394327 10.1093/biostatistics/kxx069 PMC6409418

[2] Cook D , Brown D , Alexander R , *et al*. Lessons learned from the fate of AstraZeneca's drug pipeline: a five‐dimensional framework. Nat Rev Drug Discov 2014; 13: 419–431. 24833294 10.1038/nrd4309

[3] Plenge RM , Scolnick EM , Altshuler D . Validating therapeutic targets through human genetics. Nat Rev Drug Discov 2013; 12: 581–594. 23868113 10.1038/nrd4051

[4] Lander ES , Linton LM , Birren B , *et al*. Initial sequencing and analysis of the human genome. Nature 2001; 409: 860–921. 11237011 10.1038/35057062

[5] Dietz HC . New therapeutic approaches to Mendelian disorders. N Engl J Med 2010; 363: 852–863. 20818846 10.1056/NEJMra0907180

[6] Finkel RS , Mercuri E , Darras BT , *et al*. Nusinersen versus sham control in infantile‐onset spinal muscular atrophy. N Engl J Med 2017; 377: 1723–1732. 29091570 10.1056/NEJMoa1702752

[7] Mendez MF . Early‐onset Alzheimer disease and its variants. Continuum (Minneap Minn) 2019; 25: 34–51. 30707186 10.1212/CON.0000000000000687 PMC6538053

[8] Nelson MR , Tipney H , Painter JL , *et al*. The support of human genetic evidence for approved drug indications. Nat Genet 2015; 47: 856–860. 26121088 10.1038/ng.3314

[9] King EA , Davis JW , Degner JF . Are drug targets with genetic support twice as likely to be approved? Revised estimates of the impact of genetic support for drug mechanisms on the probability of drug approval. PLoS Genet 2019; 15: e1008489. 31830040 10.1371/journal.pgen.1008489 PMC6907751

[10] Diogo D , Kurreeman F , Stahl EA , *et al*. Rare, low‐frequency, and common variants in the protein‐coding sequence of biological candidate genes from GWASs contribute to risk of rheumatoid arthritis. Am J Hum Genet 2013; 92: 15–27. 23261300 10.1016/j.ajhg.2012.11.012 PMC3542467

[11] Seddon JM , Yu Y , Miller EC , *et al*. Rare variants in CFI, C3 and C9 are associated with high risk of advanced age‐related macular degeneration. Nat Genet 2013; 45: 1366–1370. 24036952 10.1038/ng.2741 PMC3902040

[12] Khera AV , Won H‐H , Peloso GM , *et al*. Association of rare and common variation in the lipoprotein lipase gene with coronary artery disease. JAMA 2017; 317: 937–946. 28267856 10.1001/jama.2017.0972 PMC5664181

[13] Flannick J , Thorleifsson G , Beer NL , *et al*. Loss‐of‐function mutations in SLC30A8 protect against type 2 diabetes. Nat Genet 2014; 46: 357–363. 24584071 10.1038/ng.2915 PMC4051628

[14] Cochran JN , Geier EG , Bonham LW , *et al*. Non‐coding and loss‐of‐function coding variants in TET2 are associated with multiple neurodegenerative diseases. Am J Hum Genet 2020; 106: 632–645. 32330418 10.1016/j.ajhg.2020.03.010 PMC7212268

[15] Grarup N , Moltke I , Andersen MK , *et al*. Loss‐of‐function variants in ADCY3 increase risk of obesity and type 2 diabetes. Nat Genet 2018; 50: 172–174. 29311636 10.1038/s41588-017-0022-7 PMC5828106

[16] Momozawa Y , Akiyama M , Kamatani Y , *et al*. Low‐frequency coding variants in CETP and CFB are associated with susceptibility of exudative age‐related macular degeneration in the Japanese population. Hum Mol Genet 2016; 25: 5027–5034. 28173125 10.1093/hmg/ddw335

[17] 1000 Genomes Project Consortium , Auton A , Brooks LD , *et al*. A global reference for human genetic variation. Nature 2015; 526: 68–74. 26432245 10.1038/nature15393 PMC4750478

[18] McCarthy S , Das S , Kretzschmar W , *et al*. A reference panel of 64,976 haplotypes for genotype imputation. Nat Genet 2016; 48: 1279–1283. 27548312 10.1038/ng.3643 PMC5388176

[19] Lek M , Karczewski KJ , Minikel EV , *et al*. Analysis of protein‐coding genetic variation in 60,706 humans. Nature 2016; 536: 285–291. 27535533 10.1038/nature19057 PMC5018207

[20] Karczewski KJ , Francioli LC , Tiao G , *et al*. The mutational constraint spectrum quantified from variation in 141,456 humans. Nature 2020; 581: 434–443. 32461654 10.1038/s41586-020-2308-7 PMC7334197

[21] Taliun D , Harris DN , Kessler MD , *et al*. Sequencing of 53,831 diverse genomes from the NHLBI TOPMed Program. Nature 2021; 590: 290–299. 33568819 10.1038/s41586-021-03205-y PMC7875770

[22] Manolio TA , Collins FS , Cox NJ , *et al*. Finding the missing heritability of complex diseases. Nature 2009; 461: 747–753. 19812666 10.1038/nature08494 PMC2831613

[23] Kaye J , Briceño Moraia L , Mitchell C , *et al*. Access governance for biobanks: the case of the BioSHaRE‐EU cohorts. Biopreserv Biobank 2016; 14: 201–206. 27183185 10.1089/bio.2015.0124 PMC5939924

[24] Hyde CL , Nagle MW , Tian C , *et al*. Identification of 15 genetic loci associated with risk of major depression in individuals of European descent. Nat Genet 2016; 48: 1031–1036. 27479909 10.1038/ng.3623 PMC5706769

[25] Evangelou E , Warren HR , Mosen‐Ansorena D , *et al*. Genetic analysis of over 1 million people identifies 535 new loci associated with blood pressure traits. Nat Genet 2018; 50: 1412–1425. 30224653 10.1038/s41588-018-0205-x PMC6284793

[26] Jansen PR , Watanabe K , Stringer S , *et al*. Genome‐wide analysis of insomnia in 1,331,010 individuals identifies new risk loci and functional pathways. Nat Genet 2019; 51: 394–403. 30804565 10.1038/s41588-018-0333-3

[27] Liu M , Jiang Y , Wedow R , *et al*. Association studies of up to 1.2 million individuals yield new insights into the genetic etiology of tobacco and alcohol use. Nat Genet 2019; 51: 237–244. 30643251 10.1038/s41588-018-0307-5 PMC6358542

[28] Panagiotou OA , Willer CJ , Hirschhorn JN , *et al*. The power of meta‐analysis in genome‐wide association studies. Annu Rev Genomics Hum Genet 2013; 14: 441–465. 23724904 10.1146/annurev-genom-091212-153520 PMC4040957

[29] Marouli E , Graff M , Medina‐Gomez C , *et al*. Rare and low‐frequency coding variants alter human adult height. Nature 2017; 542: 186–190. 28146470 10.1038/nature21039 PMC5302847

[30] van de Bunt M , Cortes A , IGAS Consortium , *et al*. Evaluating the performance of fine‐mapping strategies at common variant GWAS loci. PLoS Genet 2015; 11: e1005535. 26406328 10.1371/journal.pgen.1005535 PMC4583479

[31] Wellcome Trust Case Control Consortium , Maller JB , McVean G , *et al*. Bayesian refinement of association signals for 14 loci in 3 common diseases. Nat Genet 2012; 44: 1294–1301. 23104008 10.1038/ng.2435 PMC3791416

[32] Thompson JR , Attia J , Minelli C . The meta‐analysis of genome‐wide association studies. Brief Bioinform 2011; 12: 259–269. 21546449 10.1093/bib/bbr020

[33] Dorsey ER , Darwin KC , Mohammed S , *et al*. Virtual research visits and direct‐to‐consumer genetic testing in Parkinson's disease. Digit Health 2015; 1: 2055207615592998. 29942542 10.1177/2055207615592998 PMC5999055

[34] Tung JY , Do CB , Hinds DA , *et al*. Efficient replication of over 180 genetic associations with self‐reported medical data. PLoS One 2011; 6: e23473. 21858135 10.1371/journal.pone.0023473 PMC3157390

[35] Ransohoff KJ , Wu W , Cho HG , *et al*. Two‐stage genome‐wide association study identifies a novel susceptibility locus associated with melanoma. Oncotarget 2017; 8: 17586–17592. 28212542 10.18632/oncotarget.15230 PMC5392271

[36] Wei WQ , Denny JC . Extracting research‐quality phenotypes from electronic health records to support precision medicine. Genome Med 2015; 7: 41. 25937834 10.1186/s13073-015-0166-y PMC4416392

[37] Cai N , Revez JA , Adams MJ , *et al*. Minimal phenotyping yields genome‐wide association signals of low specificity for major depression. Nat Genet 2020; 52: 437–447. 32231276 10.1038/s41588-020-0594-5 PMC7906795

[38] DeBoever C , Tanigawa Y , Aguirre M , *et al*. Assessing digital phenotyping to enhance genetic studies of human diseases. Am J Hum Genet 2020; 106: 611–622. 32275883 10.1016/j.ajhg.2020.03.007 PMC7212271

[39] Need AC , Goldstein DB . Next generation disparities in human genomics: concerns and remedies. Trends Genet 2009; 25: 489–494. 19836853 10.1016/j.tig.2009.09.012

[40] Bustamante CD , Burchard EG , De La Vega FM . Genomics for the world. Nature 2011; 475: 163–165. 21753830 10.1038/475163a PMC3708540

[41] Petrovski S , Goldstein DB . Unequal representation of genetic variation across ancestry groups creates healthcare inequality in the application of precision medicine. Genome Biol 2016; 17: 157. 27418169 10.1186/s13059-016-1016-y PMC4944427

[42] Popejoy AB , Fullerton SM . Genomics is failing on diversity. Nature 2016; 538: 161–164. 27734877 10.1038/538161a PMC5089703

[43] Martin AR , Kanai M , Kamatani Y , *et al*. Clinical use of current polygenic risk scores may exacerbate health disparities. Nat Genet 2019; 51: 584–591. 30926966 10.1038/s41588-019-0379-x PMC6563838

[44] Morales J , Welter D , Bowler EH , *et al*. A standardized framework for representation of ancestry data in genomics studies, with application to the NHGRI‐EBI GWAS Catalog. Genome Biol 2018; 19: 21. 29448949 10.1186/s13059-018-1396-2 PMC5815218

[45] Genovese G , Friedman DJ , Ross MD , *et al*. Association of trypanolytic ApoL1 variants with kidney disease in African Americans. Science 2010; 329: 841–845. 20647424 10.1126/science.1193032 PMC2980843

[46] Tzur S , Rosset S , Shemer R , *et al*. Missense mutations in the APOL1 gene are highly associated with end stage kidney disease risk previously attributed to the MYH9 gene. Hum Genet 2010; 128: 345–350. 20635188 10.1007/s00439-010-0861-0 PMC2921485

[47] Wohlford EM , Borrell LN , Elhawary JR , *et al*. Differential asthma odds following respiratory infection in children from three minority populations. PLoS One 2020; 15: e0231782. 32369487 10.1371/journal.pone.0231782 PMC7199930

[48] Laster M , Shen JI , Norris KC . Kidney disease among African Americans: a population perspective. Am J Kidney Dis 2018; 72: S3–S7. 30343720 10.1053/j.ajkd.2018.06.021 PMC6200351

[49] Editorial. Diversity matters. Nat Rev Genet 2019; 20: 495. 31420601 10.1038/s41576-019-0162-y

[50] Gurdasani D , Barroso I , Zeggini E , *et al*. Genomics of disease risk in globally diverse populations. Nat Rev Genet 2019; 20: 520–535. 31235872 10.1038/s41576-019-0144-0

[51] Hunter‐Zinck H , Shi Y , Li M , *et al*. Measuring genetic variation in the multi‐ethnic Million Veteran Program (MVP). *bioRxiv* 2020. 10.1101/2020.01.06.896613 [Not peer reviewed].

[52] Levey DF , Gelernter J , Polimanti R , *et al*. Reproducible genetic risk loci for anxiety: results from ∼200,000 participants in the Million Veteran Program. Am J Psychiatry 2020; 177: 223–232. 31906708 10.1176/appi.ajp.2019.19030256 PMC7869502

[53] All of Us Research Program Investigators , Denny JC , Rutter JL , *et al*. The ‘All of Us’ Research Program. N Engl J Med 2019; 381: 668–676. 31412182 10.1056/NEJMsr1809937 PMC8291101

[54] Saleheen D , Zaidi M , Rasheed A , *et al*. The Pakistan Risk of Myocardial Infarction Study: a resource for the study of genetic, lifestyle and other determinants of myocardial infarction in South Asia. Eur J Epidemiol 2009; 24: 329–338. 19404752 10.1007/s10654-009-9334-y PMC2697028

[55] Amgen to Acquire deCODE Genetics, a Global Leader in Human Genetics . Available from: https://www.amgen.com/newsroom/press-releases/2012/12/amgen-to-acquire-decode-genetics-a-global-leader-in-human-genetics. [Accessed 15 March 2021].

[56] Partners | FinnGen . Available from: https://www.finngen.fi/en/partners. [Accessed 15 March 2021].

[57] Zoledziewska M , Sidore C , Chiang CWK , *et al*. Height‐reducing variants and selection for short stature in Sardinia. Nat Genet 2015; 47: 1352–1356. 26366551 10.1038/ng.3403 PMC4627578

[58] Belbin GM , Odgis J , Sorokin EP , *et al*. Genetic identification of a common collagen disease in Puerto Ricans via identity‐by‐descent mapping in a health system. Elife 2017; 6: e25060. 28895531 10.7554/eLife.25060 PMC5595434

[59] Minster RL , Hawley NL , Su C‐T , *et al*. A thrifty variant in CREBRF strongly influences body mass index in Samoans. Nat Genet 2016; 48: 1049–1054. 27455349 10.1038/ng.3620 PMC5069069

[60] Abifadel M , Varret M , Rabès JP , *et al*. Mutations in PCSK9 cause autosomal dominant hypercholesterolemia. Nat Genet 2003; 34: 154–156. 12730697 10.1038/ng1161

[61] GAIN Collaborative Research Group , Manolio TA , Rodriguez LL , *et al*. New models of collaboration in genome‐wide association studies: the Genetic Association Information Network. Nat Genet 2007; 39: 1045–1051. 17728769 10.1038/ng2127

[62] McClay JL , Adkins DE , Aberg K , *et al*. Genome‐wide pharmacogenomic study of neurocognition as an indicator of antipsychotic treatment response in schizophrenia. Neuropsychopharmacology 2011; 36: 616–626. 21107309 10.1038/npp.2010.193 PMC3055694

[63] Carvalho‐Silva D , Pierleoni A , Pignatelli M , *et al*. Open Targets Platform: new developments and updates two years on. Nucleic Acids Res 2019; 47: D1056–D1065. 30462303 10.1093/nar/gky1133 PMC6324073

[64] Vertex and Genomics Announce Multi‐year Collaboration to Use Human Genetics and Data Science to Identify Novel Targets for Innovative Medicines ‐ GENOMICS plc . Available from: https://www.genomicsplc.com/vertex-and-genomics-collaboration/. [Accessed 15 March 2021].

[65] GSK and 23andMe Sign Agreement to Leverage Genetic Insights for the Development of Novel Medicines . Available from: https://mediacenter.23andme.com/press‐releases/gsk‐and‐23andme‐sign‐agreement‐to‐leverage‐genetic‐insights‐for‐the‐development‐of‐novel‐medicines/. [Accessed 15 March 2021].

[66] Regeneron Announces Major Collaboration to Exome Sequence UK Biobank Genetic Data More Quickly . Available from: https://www.ukbiobank.ac.uk/2018/01/regeneron‐announces‐major‐collaboration‐to‐exome‐sequence‐uk‐biobank‐genetic‐data‐more‐quickly/. [Accessed 15 March 2021].

[67] Regeneron Forms Consortium of Leading Life Sciences Companies to Accelerate Largest Widely Available ‘Big Data’ Human Sequencing Resource with UK Biobank. Available from: https://investor.regeneron.com/news‐releases/news‐release‐details/regeneron‐forms‐consortium‐leading‐life‐sciences‐companies. [Accessed 15 March 2021].

[68] Cohen J , Pertsemlidis A , Kotowski IK , *et al*. Low LDL cholesterol in individuals of African descent resulting from frequent nonsense mutations in PCSK9. Nat Genet 2005; 37: 161–165. 15654334 10.1038/ng1509

[69] Sanofi and Regeneron Announce FDA Approval of Praluent® (alirocumab) Injection , the First PCSK9 Inhibitor in the U.S., for the Treatment of High LDL Cholesterol in Adult Patients ‐ Jul 24, 2015. Available from: http://www.news.sanofi.us/2015‐07‐24‐Sanofi‐and‐Regeneron‐Announce‐FDA‐Approval‐of‐Praluent‐alirocumab‐Injection‐the‐First‐PCSK9‐Inhibitor‐in‐the‐U‐S‐for‐the‐Treatment‐of‐High‐LDL‐Cholesterol‐in‐Adult‐Patients. [Accessed 15 March 2021].

[70] FDA Approves Amgen's New Cholesterol‐Lowering Medication Repatha™ (evolocumab) . Available from: https://www.amgen.com/newsroom/press-releases/2015/08/fda-approves-amgens-new-cholesterollowering-medication-repatha-evolocumab. [Accessed 15 March 2021].

[71] FDA Approves Amgen's Repatha® (evolocumab) to Prevent Heart Attack and Stroke . Available from: https://www.amgen.com/newsroom/press‐releases/2017/12/fda‐approves‐amgens‐repatha‐evolocumab‐to‐prevent‐heart‐attack‐and‐stroke. [Accessed 15 March 2021].

[72] Ribas A , Wolchok JD . Cancer immunotherapy using checkpoint blockade. Science 2018; 359: 1350–1355. 29567705 10.1126/science.aar4060 PMC7391259

[73] Eriksson N , Tung JY , Kiefer AK , *et al*. Novel associations for hypothyroidism include known autoimmune risk loci. PLoS One 2012; 7: e34442. 22493691 10.1371/journal.pone.0034442 PMC3321023

[74] Cooper JD , Simmonds MJ , Walker NM , *et al*. Seven newly identified loci for autoimmune thyroid disease. Hum Mol Genet 2012; 21: 5202–5208. 22922229 10.1093/hmg/dds357 PMC3490518

[75] Chu X , Pan CM , Zhao SX , *et al*. A genome‐wide association study identifies two new risk loci for Graves' disease. Nat Genet 2011; 43: 897–901. 21841780 10.1038/ng.898

[76] Okada Y , Wu D , Trynka G , *et al*. Genetics of rheumatoid arthritis contributes to biology and drug discovery. Nature 2014; 506: 376–381. 24390342 10.1038/nature12873 PMC3944098

[77] Onengut‐Gumuscu S , Chen WM , Burren O , *et al*. Fine mapping of type 1 diabetes susceptibility loci and evidence for colocalization of causal variants with lymphoid gene enhancers. Nat Genet 2015; 47: 381–386. 25751624 10.1038/ng.3245 PMC4380767

[78] Liyanage UE , Law MH , Han X , *et al*. Combined analysis of keratinocyte cancers identifies novel genome‐wide loci. Hum Mol Genet 2019; 28: 3148–3160. 31174203 10.1093/hmg/ddz121 PMC6737293

[79] Elsworth B , Lyon M , Alexander T , *et al*. The MRC IEU OpenGWAS data infrastructure. *bioRxiv* 2020. 10.1101/2020.08.10.244293 [Not peer reviewed].

[80] Hemani G , Zheng J , Elsworth B , *et al*. The MR‐Base platform supports systematic causal inference across the human phenome. Elife 2018; 7: e34408. 29846171 10.7554/eLife.34408 PMC5976434

[81] Nguyen PA , Born DA , Deaton AM , *et al*. Phenotypes associated with genes encoding drug targets are predictive of clinical trial side effects. Nat Commun 2019; 10: 1579. 30952858 10.1038/s41467-019-09407-3 PMC6450952

[82] Johansson Å , Rask‐Andersen M , Karlsson T , *et al*. Genome‐wide association analysis of 350 000 Caucasians from the UK Biobank identifies novel loci for asthma, hay fever and eczema. Hum Mol Genet 2019; 28: 4022–4041. 31361310 10.1093/hmg/ddz175 PMC6969355

[83] Ferreira MA , Vonk JM , Baurecht H , *et al*. Shared genetic origin of asthma, hay fever and eczema elucidates allergic disease biology. Nat Genet 2017; 49: 1752–1757. 29083406 10.1038/ng.3985 PMC5989923

[84] Liu JZ , van Sommeren S , Huang H , *et al*. Association analyses identify 38 susceptibility loci for inflammatory bowel disease and highlight shared genetic risk across populations. Nat Genet 2015; 47: 979–986. 26192919 10.1038/ng.3359 PMC4881818

[85] Pushpakom S , Iorio F , Eyers PA , *et al*. Drug repurposing: progress, challenges and recommendations. Nat Rev Drug Discov 2019; 18: 41–58. 30310233 10.1038/nrd.2018.168

[86] Smith SM , Meyer M , Trinkley KE . Phentermine/topiramate for the treatment of obesity. Ann Pharmacother 2013; 47: 340–349. 23482732 10.1345/aph.1R501

[87] Kasperaviciute D , Catarino CB , Matarin M , *et al*. Epilepsy, hippocampal sclerosis and febrile seizures linked by common genetic variation around SCN1A. Brain 2013; 136: 3140–3150. 24014518 10.1093/brain/awt233 PMC3784283

[88] Zhu Z , Guo Y , Shi H , *et al*. Shared genetic and experimental links between obesity‐related traits and asthma subtypes in UK Biobank. J Allergy Clin Immunol 2020; 145: 537–549. 31669095 10.1016/j.jaci.2019.09.035 PMC7010560

[89] Kichaev G , Bhatia G , Loh PR , *et al*. Leveraging polygenic functional enrichment to improve GWAS power. Am J Hum Genet 2019; 104: 65–75. 30595370 10.1016/j.ajhg.2018.11.008 PMC6323418

[90] Crockett S , Schectman R , Kappelman M . Topiramate use does not reduce flares of inflammatory bowel disease: a retrospective cohort study. Am J Gastroenterol 2013; 108: S511.

[91] Cleynen I , Boucher G , Jostins L , *et al*. Inherited determinants of Crohn's disease and ulcerative colitis phenotypes: a genetic association study. Lancet 2016; 387: 156–167. 26490195 10.1016/S0140-6736(15)00465-1 PMC4714968

[92] Kamb A , Harper S , Stefansson K . Human genetics as a foundation for innovative drug development. Nat Biotechnol 2013; 31: 975–978. 24213769 10.1038/nbt.2732

[93] Sanseau P , Agarwal P , Barnes MR , *et al*. Use of genome‐wide association studies for drug repositioning. Nat Biotechnol 2012; 30: 317–320. 22491277 10.1038/nbt.2151

[94] Feagan BG , Sandborn WJ , Gasink C , *et al*. Ustekinumab as induction and maintenance therapy for Crohn's disease. N Engl J Med 2016; 375: 1946–1960. 27959607 10.1056/NEJMoa1602773

[95] Genetic Analysis of Psoriasis Consortium & the Wellcome Trust Case Control Consortium 2 , Strange A , Capon F , *et al*. A genome‐wide association study identifies new psoriasis susceptibility loci and an interaction between HLA‐C and ERAP1. Nat Genet 2010; 42: 985–990. 20953190 10.1038/ng.694 PMC3749730

[96] Franke A , McGovern DP , Barrett JC , *et al*. Genome‐wide meta‐analysis increases to 71 the number of confirmed Crohn's disease susceptibility loci. Nat Genet 2010; 42: 1118–1125. 21102463 10.1038/ng.717 PMC3299551

[97] Nabirotchkin S , Peluffo AE , Rinaudo P , *et al*. Next‐generation drug repurposing using human genetics and network biology. Curr Opin Pharmacol 2020; 51: 78–92. 31982325 10.1016/j.coph.2019.12.004

[98] Morris JA , Kemp JP , Youlten SE , *et al*. An atlas of genetic influences on osteoporosis in humans and mice. Nat Genet 2019; 51: 258–266. 30598549 10.1038/s41588-018-0302-x PMC6358485

[99] Kemp JP , Morris JA , Medina‐Gomez C , *et al*. Identification of 153 new loci associated with heel bone mineral density and functional involvement of GPC6 in osteoporosis. Nat Genet 2017; 49: 1468–1475. 28869591 10.1038/ng.3949 PMC5621629

[100] Torkamani A , Wineinger NE , Topol EJ . The personal and clinical utility of polygenic risk scores. Nat Rev Genet 2018; 19: 581–590. 29789686 10.1038/s41576-018-0018-x

[101] Lewis CM , Vassos E . Polygenic risk scores: from research tools to clinical instruments. Genome Med 2020; 12: 44. 32423490 10.1186/s13073-020-00742-5 PMC7236300

[102] Desikan RS , Fan CC , Wang Y , *et al*. Genetic assessment of age‐associated Alzheimer disease risk: development and validation of a polygenic hazard score. PLoS Med 2017; 14: e1002258. 28323831 10.1371/journal.pmed.1002258 PMC5360219

[103] Abraham G , Malik R , Yonova‐Doing E , *et al*. Genomic risk score offers predictive performance comparable to clinical risk factors for ischaemic stroke. Nat Commun 2019; 10: 5819. 31862893 10.1038/s41467-019-13848-1 PMC6925280

[104] Fontanillas P , Alipanahi B , Furlotte NA , *et al*. Disease risk scores for skin cancers. Nat Commun 2021; 12: 160. 33420020 10.1038/s41467-020-20246-5 PMC7794415

[105] Khera AV , Chaffin M , Aragam KG , *et al*. Genome‐wide polygenic scores for common diseases identify individuals with risk equivalent to monogenic mutations. Nat Genet 2018; 50: 1219–1224. 30104762 10.1038/s41588-018-0183-z PMC6128408

[106] Miki Y , Swensen J , Shattuck‐Eidens D , *et al*. A strong candidate for the breast and ovarian cancer susceptibility gene BRCA1. Science 1994; 266: 66–71. 7545954 10.1126/science.7545954

[107] Wooster R , Bignell G , Lancaster J , *et al*. Identification of the breast cancer susceptibility gene BRCA2. Nature 1995; 378: 789–792. 8524414 10.1038/378789a0

[108] Fahed AC , Wang M , Homburger JR , *et al*. Polygenic background modifies penetrance of monogenic variants for tier 1 genomic conditions. Nat Commun 2020; 11: 3635. 32820175 10.1038/s41467-020-17374-3 PMC7441381

[109] Stocker H , Perna L , Weigl K , *et al*. Prediction of clinical diagnosis of Alzheimer's disease, vascular, mixed, and all‐cause dementia by a polygenic risk score and APOE status in a community‐based cohort prospectively followed over 17 years. Mol Psychiatr 2020. https://www.nature.com/articles/s41380-020-0764-y 10.1038/s41380-021-01311-x PMC8758469 34599279

[110] Knevel R , le Cessie S , Terao CC , *et al*. Using genetics to prioritize diagnoses for rheumatology outpatients with inflammatory arthritis. Sci Transl Med 2020; 12: eaay1548. 32461333 10.1126/scitranslmed.aay1548 PMC7341896

[111] Kiernan MC , Vucic S , Talbot K , *et al*. Improving clinical trial outcomes in amyotrophic lateral sclerosis. Nat Rev Neurol 2021; 17: 104–118. 33340024 10.1038/s41582-020-00434-z PMC7747476

[112] Udler MS , McCarthy MI , Florez JC , *et al*. Genetic risk scores for diabetes diagnosis and precision medicine. Endocr Rev 2019; 40: 1500–1520. 31322649 10.1210/er.2019-00088 PMC6760294

[113] de Carvalho M , Swash M . Can selection of rapidly progressing patients shorten clinical trials in amyotrophic lateral sclerosis? Arch Neurol 2006; 63: 557–560. 16606769 10.1001/archneur.63.4.557

[114] Eslam M , George J . Genetic contributions to NAFLD: leveraging shared genetics to uncover systems biology. Nat Rev Gastroenterol Hepatol 2020; 17: 40–52. 31641249 10.1038/s41575-019-0212-0

[115] Harrison SA , Abdelmalek MF , Caldwell S , *et al*. Simtuzumab is ineffective for patients with bridging fibrosis or compensated cirrhosis caused by nonalcoholic steatohepatitis. Gastroenterology 2018; 155: 1140–1153. 29990488 10.1053/j.gastro.2018.07.006

[116] Natarajan P , Young R , Stitziel NO , *et al*. Polygenic risk score identifies subgroup with higher burden of atherosclerosis and greater relative benefit from statin therapy in the primary prevention setting. Circulation 2017; 135: 2091–2101. 28223407 10.1161/CIRCULATIONAHA.116.024436 PMC5484076

[117] Damask A , Steg PG , Schwartz GG , *et al*. Patients with high genome‐wide polygenic risk scores for coronary artery disease may receive greater clinical benefit from alirocumab treatment in the ODYSSEY OUTCOMES Trial. Circulation 2020; 141: 624–636. 31707832 10.1161/CIRCULATIONAHA.119.044434

[118] Kogelman LJA , Esserlind AL , Francke Christensen A , *et al*. Migraine polygenic risk score associates with efficacy of migraine‐specific drugs. Neurol Genet 2019; 5: e364. 31872049 10.1212/NXG.0000000000000364 PMC6878840

[119] Khan Z , Di Nucci F , Kwan A , *et al*. Polygenic risk for skin autoimmunity impacts immune checkpoint blockade in bladder cancer. Proc Natl Acad Sci U S A 2020; 117: 12288–12294. 32430334 10.1073/pnas.1922867117 PMC7275757

[120] Nalls MA , Blauwendraat C , Vallerga CL , *et al*. Identification of novel risk loci, causal insights, and heritable risk for Parkinson's disease: a meta‐analysis of genome‐wide association studies. Lancet Neurol 2019; 18: 1091–1102. 31701892 10.1016/S1474-4422(19)30320-5 PMC8422160

[121] Hill AB . The environment and disease: association or causation? Proc R Soc Med 1965; 58: 295–300. 14283879 10.1177/003591576505800503 PMC1898525

[122] Tan MMX , Lawton MA , Jabbari E , *et al*. Genome‐wide association studies of cognitive and motor progression in Parkinson's disease. Mov Disord 2021; 36: 424–433. 33111402 10.1002/mds.28342 PMC9053517

[123] Severe Covid‐19 GWAS Group , Ellinghaus D , Degenhardt F , *et al*. Genomewide association study of severe Covid‐19 with respiratory failure. N Engl J Med 2020; 383: 1522–1534. 32558485 10.1056/NEJMoa2020283 PMC7315890

[124] Shelton JF , Shastri AJ , Ye C , *et al*. Trans‐ethnic analysis reveals genetic and non‐genetic associations with COVID‐19 susceptibility and severity. *medRxiv* 2020. 10.1101/2020.09.04.20188318 [Not peer reviewed].

[125] Roberts GHL , Park DS , Coignet MV , *et al*. AncestryDNA COVID‐19 host genetic study identifies three novel loci. *medRxiv* 2020. 10.1101/2020.10.06.20205864 [Not peer reviewed].

[126] Pairo‐Castineira E , Clohisey S , Klaric L , *et al*. Genetic mechanisms of critical illness in COVID‐19. Nature 2021; 591: 92–98. 33307546 10.1038/s41586-020-03065-y

[127] Lee PH , Lee C , Li X , *et al*. Principles and methods of in‐silico prioritization of non‐coding regulatory variants. Hum Genet 2018; 137: 15–30. 29288389 10.1007/s00439-017-1861-0 PMC5892192

[128] ENCODE Project Consortium . An integrated encyclopedia of DNA elements in the human genome. Nature 2012; 489: 57–74. 22955616 10.1038/nature11247 PMC3439153

[129] GTEx Consortium . The Genotype‐Tissue Expression (GTEx) project. Nat Genet 2013; 45: 580–585. 23715323 10.1038/ng.2653 PMC4010069

[130] Gaj T , Gersbach CA , Barbas CF 3rd. ZFN, TALEN, and CRISPR/Cas‐based methods for genome engineering. Trends Biotechnol 2013; 31: 397–405. 23664777 10.1016/j.tibtech.2013.04.004 PMC3694601

[131] Manguso RT , Pope HW , Zimmer MD , *et al*. In vivo CRISPR screening identifies Ptpn2 as a cancer immunotherapy target. Nature 2017; 547: 413–418. 28723893 10.1038/nature23270 PMC5924693

[132] Behan FM , Iorio F , Picco G , *et al*. Prioritization of cancer therapeutic targets using CRISPR‐Cas9 screens. Nature 2019; 568: 511–516. 30971826 10.1038/s41586-019-1103-9

[133] Chan EM , Shibue T , McFarland JM , *et al*. WRN helicase is a synthetic lethal target in microsatellite unstable cancers. Nature 2019; 568: 551–556. 30971823 10.1038/s41586-019-1102-x PMC6580861

[134] Gupta RM , Hadaya J , Trehan A , *et al*. A genetic variant associated with five vascular diseases is a distal regulator of endothelin‐1 gene expression. Cell 2017; 170: 522–533.e15. 28753427 10.1016/j.cell.2017.06.049 PMC5785707

[135] Ott J . Analysis of Human Genetic Linkage (3rd edn). Johns Hopkins University Press: London, 1999.

[136] Cooper SE , Schwartzentruber J , Bello E , *et al*. Screening for functional transcriptional and splicing regulatory variants with GenIE. Nucleic Acids Res 2020; 48: e131. 33152068 10.1093/nar/gkaa960 PMC7736817

[137] Hanna RE , Hegde M , Fagre CR , *et al*. Massively parallel assessment of human variants with base editor screens. Cell 2021; 184: 1064–1080.e20. 33606977 10.1016/j.cell.2021.01.012

[138] Gasperini M , Hill AJ , McFaline‐Figueroa JL , *et al*. A genome‐wide framework for mapping gene regulation via cellular genetic screens. Cell 2019; 176: 1516. 30849375 10.1016/j.cell.2019.02.027

[139] Schraivogel D , Gschwind AR , Milbank JH , *et al*. Targeted Perturb‐seq enables genome‐scale genetic screens in single cells. Nat Methods 2020; 17: 629–635. 32483332 10.1038/s41592-020-0837-5 PMC7610614

[140] Forgetta V , Keller‐Baruch J , Forest M , *et al*. Development of a polygenic risk score to improve screening for fracture risk: a genetic risk prediction study. PLoS Med 2020; 17: e1003152. 32614825 10.1371/journal.pmed.1003152 PMC7331983

[141] Mosley JD , Gupta DK , Tan J , *et al*. Predictive accuracy of a polygenic risk score compared with a clinical risk score for incident coronary heart disease. JAMA 2020; 323: 627–635. 32068817 10.1001/jama.2019.21782 PMC7042849

[142] Martin AR , Gignoux CR , Walters RK , *et al*. Human demographic history impacts genetic risk prediction across diverse populations. Am J Hum Genet 2020; 107: 788–789. 33007199 10.1016/j.ajhg.2020.08.020 PMC7536609

[143] Dudbridge F . Power and predictive accuracy of polygenic risk scores. PLoS Genet 2013; 9: e1003348. 23555274 10.1371/journal.pgen.1003348 PMC3605113

[144] Lei SF , Yang TL , Tan LJ , *et al*. Genome‐wide association scan for stature in Chinese: evidence for ethnic specific loci. Hum Genet 2009; 125: 1–9. 19030899 10.1007/s00439-008-0590-9 PMC2730511

[145] Cho YS , Go MJ , Kim YJ , *et al*. A large‐scale genome‐wide association study of Asian populations uncovers genetic factors influencing eight quantitative traits. Nat Genet 2009; 41: 527–534. 19396169 10.1038/ng.357

[146] Okada Y , Kamatani Y , Takahashi A , *et al*. A genome‐wide association study in 19 633 Japanese subjects identified LHX3‐QSOX2 and IGF1 as adult height loci. Hum Mol Genet 2010; 19: 2303–2312. 20189936 10.1093/hmg/ddq091

[147] Akiyama M , Ishigaki K , Sakaue S , *et al*. Characterizing rare and low‐frequency height‐associated variants in the Japanese population. Nat Commun 2019; 10: 4393. 31562340 10.1038/s41467-019-12276-5 PMC6764965

[148] Nagy R , Boutin TS , Marten J , *et al*. Exploration of haplotype research consortium imputation for genome‐wide association studies in 20,032 Generation Scotland participants. Genome Med 2017; 9: 23. 28270201 10.1186/s13073-017-0414-4 PMC5339960

[149] Lango Allen H , Estrada K , Lettre G , *et al*. Hundreds of variants clustered in genomic loci and biological pathways affect human height. Nature 2010; 467: 832–838. 20881960 10.1038/nature09410 PMC2955183

[150] Wood AR , Esko T , Yang J , *et al*. Defining the role of common variation in the genomic and biological architecture of adult human height. Nat Genet 2014; 46: 1173–1186. 25282103 10.1038/ng.3097 PMC4250049

[151] Wojcik GL , Graff M , Nishimura KK , *et al*. Genetic analyses of diverse populations improves discovery for complex traits. Nature 2019; 570: 514–518. 31217584 10.1038/s41586-019-1310-4 PMC6785182

[152] Bycroft C , Freeman C , Petkova D , *et al*. The UK Biobank resource with deep phenotyping and genomic data. Nature 2018; 562: 203–209. 30305743 10.1038/s41586-018-0579-z PMC6786975

[153] Wang H , Zhang F , Zeng J , *et al*. Genotype‐by‐environment interactions inferred from genetic effects on phenotypic variability in the UK Biobank. Sci Adv 2019; 5: eaaw3538. 31453325 10.1126/sciadv.aaw3538 PMC6693916

[154] Yengo L , Sidorenko J , Kemper KE , *et al*. Meta‐analysis of genome‐wide association studies for height and body mass index in ∼700000 individuals of European ancestry. Hum Mol Genet 2018; 27: 3641–3649. 30124842 10.1093/hmg/ddy271 PMC6488973

[155] Gudbjartsson DF , Walters GB , Thorleifsson G , *et al*. Many sequence variants affecting diversity of adult human height. Nat Genet 2008; 40: 609–615. 18391951 10.1038/ng.122

[156] Fang H , Hui Q , Lynch J , *et al*. Harmonizing genetic ancestry and self‐identified race/ethnicity in genome‐wide association studies. Am J Hum Genet 2019; 105: 763–772. 31564439 10.1016/j.ajhg.2019.08.012 PMC6817526

