# Supplementary material for: Advancing drug discovery using the power of the human genome
Source: J Pathol. 2021 Apr 9;254(4):418–29. doi: 10.1002/path.5664 (PMC8251523; doi:10.1002/path.5664)
Supplement: Supplementary file 1 — Table S1. Height GWAS used in Figure 1A [file PATH-254-418-s001.docx]

**Advancing drug discovery using the power of the human genome**

K Heilbron, SV Mozaffari *et al. J Pathol* DOI: 10.1002/path.5664

**Table S1. Height GWAS used in Figure 1A**

| **Study** | **Identifier** | **Population** | **N** | **Hits** | **Notes** |
| --- | --- | --- | --- | --- | --- |
| Lei 2009 [144] | GCST000272 | East Asian | 618 | 0 | Abstract states that no SNP had P < 5e-8 |
| Cho 2009 [145] | GCST000380 | East Asian | 8,842 | 2 | Number of hits taken from Table 1 |
| Okada 2010 [146] | GCST000611 | East Asian | 19,633 | 8 | Number of hits taken from the Abstract |
| Akiyama 2019 [147] | GCST008839 | East Asian | 159,095 | 363 | 363 primary hits (plus 246 conditional hits) |
| Nagy 2017 [148] | GCST004212 | European | 19,965 | 26 | Number of hits taken from Table 1 |
| Lango Allen 2010 [149] | GCST000817 | European | 133,653 | 207 | Discovered 207 loci, 180 survived meta-analysis with fresh samples, and there were 19 additional conditional hits |
| Wood 2014 [150] | GCST002647 | European | 253,288 | 423 | 423 loci containing 697 independent hits |
| Wojcik 2019 [151] | GCST008040 | European | 303,288 | 519 | This study conducted both a European-only meta-analysis and a multi-ethnic meta-analysis. Numbers taken from Extended Data Table 2. |
| Bycroft 2018 [152] | GCST008974 | European | 343,321 | 1,186 | Numbers were summed from Figure 4. Regressions were performed using BOLT-LMM. |
| Wang 2019 [153] | GCST009142 | European | 347,086 | 1,063 | Number of hits taken from Table 1. Note that they used a more stringent P value threshold (2e-9) due to testing multiple traits elsewhere in the paper, so the number of hits presented here is conservative. |
| Yengo 2018 [154] | GCST006901 | European | 693,529 | 2,388 | Number of (primary) hits taken from the Results section. Note that there were 3,290 independent hits in the conditional analysis. |
| Gudbjartsson 2008 [155] | GCST000175 | Multi-ethnic | 30,968 | 23 | Number of hits taken from Table 1 after removing those with P > 5e-8. The authors state that the effective sample size was 27,224. |
| Wojcik 2019 [151] | GCST008053 | Multi-ethnic | 303,288 | 487 | Numbers from Extended Data Table 2 |
| Fang 2019 [156] | GCST008904 | Multi-ethnic | 342,883 | 416 | Number of hits taken from the “GWAS for Height in MVP” section. Sample size taken from the Methods section. |

Study = surname of the first author and the year of publication [reference numbers refer to the main text list]. Identifier = GWAS catalog study identifier, N = sample size, Hits = number of loci with P < 5e-8, Notes = additional information on how relevant information was extracted from these publications.
